# The Role of the Adhesion Receptor CD146 and Its Soluble Form in Human Embryo Implantation and Pregnancy

**DOI:** 10.3389/fimmu.2021.711394

**Published:** 2021-08-26

**Authors:** Sylvie Bouvier, Elise Kaspi, Ahmad Joshkon, Odile Paulmyer-Lacroix, Marie-Dominique Piercecchi-Marti, Akshita Sharma, Aurélie S. Leroyer, Alexandrine Bertaud, Jean-Christophe Gris, Françoise Dignat-George, Marcel Blot-Chabaud, Nathalie Bardin

**Affiliations:** ^1^Department of Hematology, Nîmes University Hospital, Nîmes, France; ^2^Faculty of Pharmaceutical and Biological Sciences, University of Montpellier, Montpellier, France; ^3^UA11 Institute Desbrest of Epidemiology and Public Health, INSERM, Univ Montpellier, Montpellier, France; ^4^Aix Marseille Univ, APHM, INSERM, MMG, Hôpital la Timone, Service de Biologie Cellulaire, Marseille, France; ^5^Aix-Marseille Univ, INSERM, INRAE, C2VN, Marseille, France; ^6^Aix Marseille Univ, APHM, Hôpital la Conception, Laboratory of Histology-Embryology/Biology of Reproduction, Marseille, France; ^7^Aix Marseille Univ, APHM, CNRS, EFS, ADES, Hôpital La Timone, Forensic Department, Marseille, France; ^8^Department of Stem Cell and Regenerative Medicine, Centre for Interdisciplinary Research, DY Patil University, Kolhapur, India; ^9^I.M. Sechenov First Moscow State Medical University, Moscow, Russia; ^10^Aix Marseille Univ, APHM, INSERM, INRAE, C2VN, Hôpital la Conception, Laboratoire d’Hématologie, Marseille, France; ^11^Aix Marseille Univ, APHM, INSERM, INRAE, C2VN, Hôpital la Conception, Laboratoire d’Immunologie, Marseille, France

**Keywords:** biomarker, CD146/sCD146, fertility, implantation, pregnancy, preeclampsia

## Abstract

CD146 is an adhesion molecule essentially located in the vascular system, which has been described to play an important role in angiogenesis. A soluble form of CD146, called sCD146, is detected in the bloodstream and is known as an angiogenic factor. During placental development, CD146 is selectively expressed in extravillous trophoblasts. A growing body of evidence shows that CD146 and, in particular, sCD146, regulate extravillous trophoblasts migration and invasion both *in vitro* and *in vivo*. Hereby, we review expression and functions of CD146/sCD146 in the obstetrical field, mainly in pregnancy and in embryo implantation. We emphasized the relevance of quantifying sCD146 in the plasma of pregnant women or in embryo supernatant in the case of *in vitro* fertilization (IVF) to predict pathological pregnancy such as preeclampsia or implantation defect. This review will also shed light on some major results that led us to define CD146/sCD146 as a biomarker of placental development and paves the way toward identification of new therapeutic targets during implantation and pregnancy.

## Introduction

Successful embryo implantation, placentation, and subsequent gestation depend on complex coordinated interactions between maternal and fetal tissues. Invasion of trophoblasts into the deciduum and the myometrium to subsequently establish the uteroplacental vasculature is indispensable for an effective pregnancy (1). During the early phase of pregnancy, cytotrophoblasts differentiate into two major cell lineages, the syncytiotrophoblasts and the extravillous trophoblasts (EVTs) that form endovascular and interstitial invasive trophoblasts. The interstitial invasive trophoblasts invade uterine tissue and anchor the placenta to the uterus, while the endovascular invasive trophoblasts migrate to the maternal uterine spiral arteries transforming them into large diameter conduit vessels of low resistance to establish the uteroplacental circulation (2). Trophoblasts invasion is regulated by not only various angiogenic growth factors but also adhesion molecules and oxygen concentration (3). Thus, migrating EVTs secrete the angiogenic growth factors: vascular endothelial growth factor (VEGF) and the soluble form of CD146 (sCD146), which promote angiogenesis in the decidua.

CD146, often referred to as MUC18, melanoma cell adhesion molecule (MCAM, Mel-CAM), is an adhesion molecule belonging to the immunoglobulin superfamily. This transmembrane glycoprotein of 113 kDa is present in several isoforms, short and long membrane isoforms (4), and a soluble form (sCD146) generated by a membrane proteolysis *via* metalloproteinases (5). CD146 has a preferential localization at endothelial cell junctions (6) and is expressed not only on all types of human endothelial cells but also on other cell types such as TH17 lymphocytes (7, 8), EVTs (9), and cancers of various origins as melanoma cells or malignant mesothelioma cells (10–13). Soluble CD146 is detected in the supernatant of cultured cells and in human sera from healthy patients and patients with pathologies associated with vascular disorders (14).

The angiogenic function of CD146 and its soluble form under both physiological and pathological conditions including cancers is well-documented and has been recently reviewed (15). A growing body of evidence shows that CD146, and in particular its soluble form, regulates obstetrical angiogenesis (16–18). This review will summarize our current understanding of CD146/sCD146 contribution in obstetrics.

## Expression and Localization of CD146 at the Human Materno-Fetal Interface

CD146 is only expressed in uteri of pregnant women and is totally absent in uteri of non-pregnant women (17). Its expression on the placental villi exhibits a pattern of spatial selectivity, progressively increasing in the zone of anchoring between the villi and the deciduum, allowing the attachment of the placenta to the uterine wall (19). Indeed, after implantation, the outermost cell layer of the blastocyst, the trophectoderm, gives rise to mononuclear cytotrophoblasts forming placental villi through branching morphogenesis. Then, syncytiotrophoblasts are generated by cell fusion of villous cytotrophoblasts. Whereas syncytiotrophoblasts of floating villi represent the transport units of the human placenta, anchoring villi of the placental basal plate form another differentiated trophoblast type, the so-called invasive extravillous trophoblast (20). Thus, CD146 is mainly expressed on intermediate, a subtype of trophoblasts morphologically and functionally between syncytiotrophoblasts and cytotrophoblasts (21), and extravillous trophoblasts that are characterized by their high migrative and invasive capabilities. CD146 expression on intermediate trophoblasts facilitates their binding to uterine smooth muscle cells, which limits the extension of the trophoblast migration zone to the site of implantation (22).

Besides, CD146 is highly expressed in the endometrium and cumulus–oocyte complex and in the trophectoderm and inner cell mass of the blastocyst (23). Moreover, Liu et al. showed that CD146 is potently upregulated both in receptive maternal uteri and invasive embryonic trophoblasts only during early stages of pregnancy, which progressively fades afterwards (17). These data reinforce the importance of CD146 at the human materno-fetal interface.

**Figure 1:**
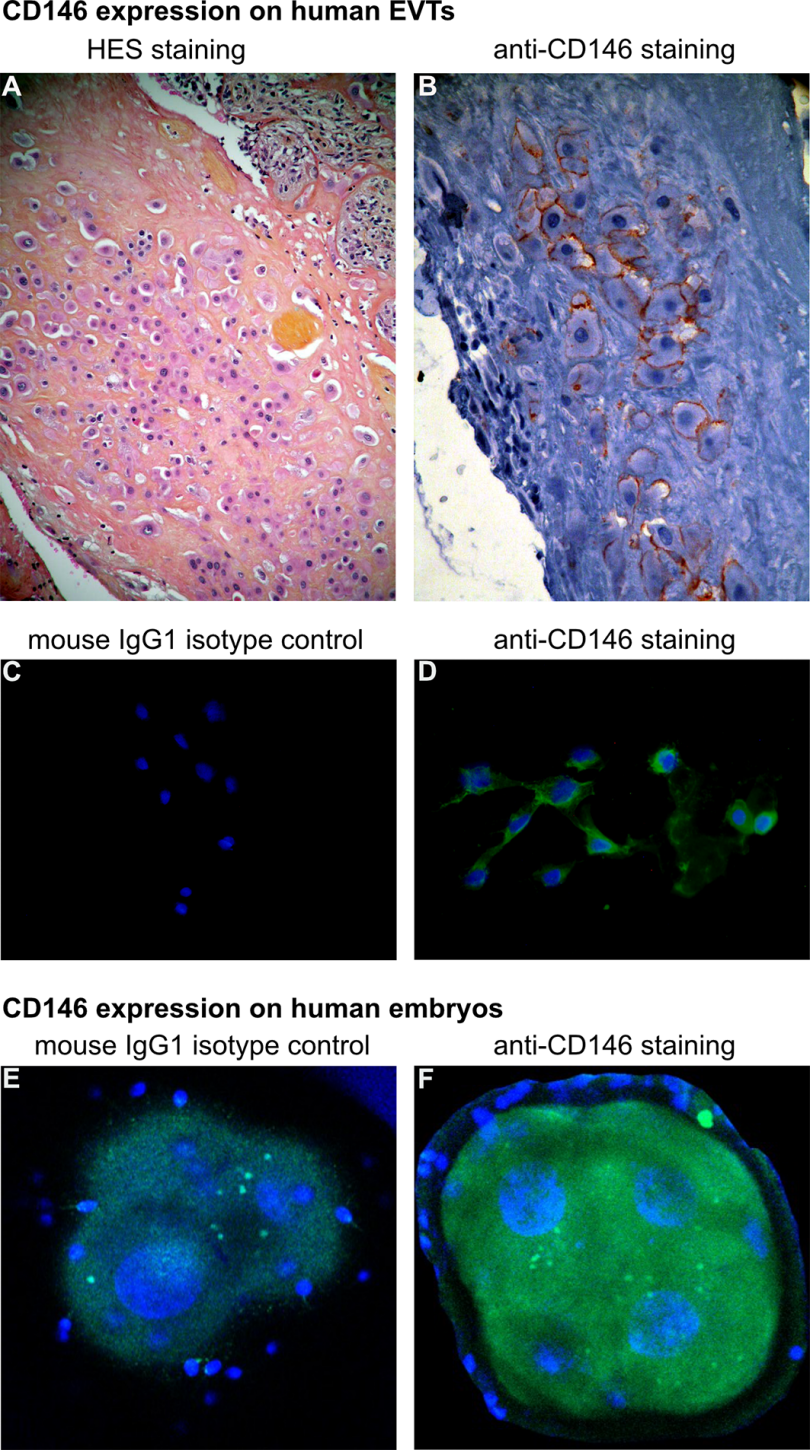
CD146 expression on human extravillous trophoblasts (EVTs) and embryos. **(A, B)** Characterization of EVTs in human placenta, from a normal term pregnancy. **(A)** EVTs are stained with hematoxylin and eosin (H&E) (objective 10×). **(B)** Immunohistochemistry with avidin–biotin–peroxidase complex, with CD146 antibody (monoclonal mouse antihuman, BioCytex S-Endo 1S-Endo1) (objective 20×). **(C, D)** Expression of CD146 on EVTs outgrowth from human placental villous explants culture (*ex vivo* model) [for model, see Kaspi et al., (18)]. **(C)** Cells were labeled with mouse IgG1 isotype control and **(D)** anti-CD146 antibody (Novocastra-Leica N1238). 4′,6-Diamidino-2-phenylindole (DAPI) was used to counterstain DNA (objective 20×). **(E, F)** Expression of CD146 on early embryo stage (day 2): immunostaining of **(E)** mouse IgG1 isotype control and **(F)** anti-CD146 (S-Endo1). DAPI was used to counterstain DNA. [Picture is from Bouvier et al, (24)] (objective 63×).

**Figure 1** highlights CD146 expression on human embryos and EVTs from human placenta.

## Strategic Roles of CD146 in Obstetrics

Extravillous trophoblasts play a crucial role in establishing the fetal–maternal circulation, essentially by invading the deciduum and remodeling the uterine spiral arteries. During early phases of pregnancy, CD146 appears to play an essential role in regulating intermediate and extravillous trophoblasts invasion and migration to ensure adequate embryo implantation and vascularization (16, 17). Indeed, *in vitro* and *in vivo* experiments showed that anti-CD146 blocking antibodies prevent embryo implantation by inhibiting MMP-2 and MMP-9 collagenase activity, impeding trophoblast proliferation and blocking deciduum angiogenesis and vascularization (17).

However, there is scarcity of knowledge concerning factors that regulate CD146 expression on extravillous trophoblasts. Data revealed that reduced expression of CD146 on the placenta is directly linked to preeclampsia, a pregnancy complication related to high blood pressure (16). Besides, it has been shown that during placentation, the deciduum cells promote CD146 expression on extravillous trophoblasts and enhance their differentiation by activating cAMP-dependent signaling pathway (25).

## What About the Soluble Form of CD146 in Obstetrics?

Recent studies demonstrated the role of sCD146 as a regulator of trophoblast migration and potent stimulator of placental vascularization (18). *In vitro* experiments performed on EVTs cell line, HTR8/SVneo, showed that sCD146 inhibits their migration, invasion, and ability to form pseudo-capillary tubes in Matrigel. Likewise, *ex vivo* experiments on placental villous explants showed that sCD146 suppressed outgrowth and migration of EVTs. Accordingly, sCD146 was identified as a negative regulator of EVTs migration (18). Kaspi et al. (18) additionally investigated the *in vivo* functions of sCD146 in the placentation and fertility in a rat model. We showed that rats treated with recombinant sCD146 exhibit not only diminished number of pregnancies but also decreased number of embryos. Interestingly, histological studies performed on placenta evidenced reduced migration of Glycogen cells (GC), analogue to human extravillous cytotrophoblasts, in treated rats as compared to the controls (18). These results corroborate the inhibitory effect of sCD146 on extravillous trophoblasts migration. Therefore, it was proposed that sCD146 acts early in gestation, probably during implantation step and/or during placental development to regulate extravillous trophoblasts invasion.

## How Does it Work?

Similarities between physiological blastocyst implantation and pathological neoplasm invasion are reported (26). Indeed, CD146 was first proposed as a marker of melanoma metastasis (27), but later, Liu et al. showed that CD146 promoted trophoblast invasion, implantation, and placentation (17). It is now well-recognized that the soluble form of multiple adhesion molecules implicated in pregnancy can modulate the function of the membrane protein, the best-known model being the effect of the soluble form of the vascular endothelial growth factor receptor-1 (VEGFR1), also called sFlt1 (28). In line with these data, it is assumed that sCD146 prevents the interaction between CD146 and its binding partners that act to induce trophoblasts invasion, differentiation, and placentation.

CD146 and galectin-1 (Gal1) are proangiogenic factors that are expressed in EVTs (29). To elucidate sCD146 mechanism of action on trophoblasts, HTR8/SVneo cells were treated with Gal1 and sCD146 (submitted manuscript). Results showed that these two molecules exerted an opposite effect on cell migration: sCD146 significantly decreasing HTR8/SVneo cells migration while Gal1 potentiating it. Of importance, sCD146 blocked Gal1-induced migratory effects on trophoblasts by inhibiting its secretion. This suggests that sCD146 acts as a ligand trap and antagonizes the effects and signaling mediated by membrane CD146. Besides, *in vitro* experiments using blocking anti-CD146 antibody or knocking down VEGFR2 inhibited Gal1-induced trophoblasts migration. Thus, it is proposed that the binding of Gal1 to CD146 on trophoblasts activates VEGFR2 signaling pathway.

In addition, the cognate interaction between VEGF and VEGFR2 is known to generate reactive oxygen species *via* NADPH-oxidase complex (NOX4) and Rac1 (protein of the Rho family) (30). As oxygen concentration is implicated in modifying trophoblasts invasion and differentiation (31), future work will validate if oxygen controls CD146 expression on trophoblasts or sCD146 generation.

## Soluble CD146: A Potential Candidate Biomarker to Predict Pathological Pregnancies or Implantation Defects?

Under physiological conditions, the serum concentration of sCD146 progressively decreases throughout normal pregnancy, a result confirmed in two independent cohorts of patients (18, Bouvier et al., submitted manuscript). However, sCD146 concentration was found to be elevated in patients with at least one unexplained fetal loss as compared to women with at least one viable child (32). Bouvier et al. have quantified sCD146 plasma concentration in a cohort of women with placental-mediated pregnancy complications. They found sCD146 to be upregulated (21%) in women with preeclampsia as compared to women with normal pregnancy (submitted manuscript; clinicaltrials.gov identifier: NCT 01736826).

In view of these results, sCD146 may represent an attractive biomarker to assess abnormality in placental vascular development and constitute a potential predictive biomarker to discriminate between normal pregnancies and pathological ones.

In addition, since CD146 is detected only at early developmental stages of human embryos (day 2) and as *in vitro* fertilized eggs secreted sCD146 into their culture media, it has been proposed that sCD146 may act as a biomarker in reproductive medicine for evaluating embryos’ implantation potential. Data revealed that high concentration of sCD146 in embryo culture media is associated with lower implantation potentials (24). The sensitivity analysis performed on single embryo transfer showed that the optimal sCD146 concentration for a successful embryo implantation is just under 1,164 pg/ml. Beyond this value, the implantation rate decreased significantly [9% with sCD146 levels >1,164 pg/ml *vs*. 22% with sCD146 levels ≤1,164 pg/ml (24)]. Therefore, sCD146 was considered as an innovative biomarker for selecting the best embryos during *in vitro* fertilization (IVF).

## Conclusion

**Figure 2:**
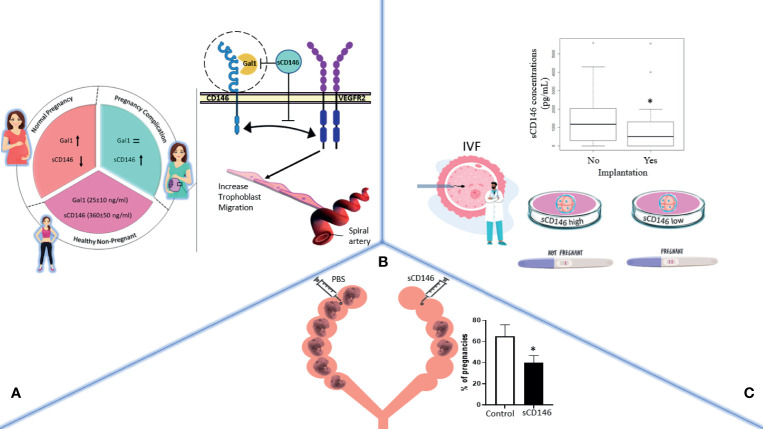
The general effects of CD146/sCD146 in obstetrics are summarized in three illustrations. **(A)** In pregnancy, sCD146 and galectin 1 (Gal1) plasma concentration varies throughout the different stages of pregnancy. In case of pregnancy complications, sCD146 concentration surges, which in turn hinders Gal1/membrane CD146 interaction, mutually known to activate trophoblasts migration toward the spiral arteries. **(B)** In implantation, sCD146 injection into female pregnant rats significantly decreases the number of embryos per litter. **(C)** In IVF, *in vitro* fertilized eggs producing high concentrations of sCD146 into their culture media fail implantation when transferred into the uterus, a boxplot of sCD146 concentrations between implanted (Yes, n = 37) and non-implanted embryos (No, n = 185) (*p* = 0.024) is shown. *p < 0.05.

In this review, we provide an overview of the diverse roles of CD146/sCD146 in human embryo implantation and pregnancy as summarized in **Figure 2**. CD146/sCD146 can be proposed as a biomarker of placental development. Taking into account the functional studies of CD146/sCD146 performed on EVTs and the sCD146 seric concentration in placental-mediated pregnancy complications, the description of the expression and functions of CD146/sCD146 also paves the way to the development of new therapeutic agents targeting CD146/sCD146 in obstetrics complications.

## Author Contributions

SB and EK contribute equally to this work and performed the majority of experiments. AJ made experiments and contribute to the redaction of the manuscript. OP-L and MP-M analyzed embryo and obstetrical data. AL and AB analyzed data concerning mechanism. J-CG and FD-G contribute to the reviewing of the manuscript. MB-C contribute to the writing. NB designed the studies and wrote the review. All authors contributed to the article and approved the submitted version.

## Conflict of Interest

The authors declare that the research was conducted in the absence of any commercial or financial relationships that could be construed as a potential conflict of interest.

## Publisher’s Note

All claims expressed in this article are solely those of the authors and do not necessarily represent those of their affiliated organizations, or those of the publisher, the editors and the reviewers. Any product that may be evaluated in this article, or claim that may be made by its manufacturer, is not guaranteed or endorsed by the publisher.
